# Preliminary Characterization of the Transcriptional Response of the Porcine Intestinal Cell Line IPEC-J2 to Enterotoxigenic *Escherichia coli*, *Escherichia coli*, and *E. coli* Lipopolysaccharide

**DOI:** 10.1155/2010/469583

**Published:** 2010-12-29

**Authors:** Marisa M. Geens, Theo A. Niewold

**Affiliations:** Division of Livestock Nutrition Quality, Department of Biosystems, K.U.Leuven, 3001 Heverlee, Belgium

## Abstract

IPEC-J2, a promising *in vitro* model system, is not well characterized especially on the transcriptional level, in contrast to human counterparts. The aim of this study was to characterize the gene expression in IPEC-J2 cells when coincubated with enterotoxigenic *Escherichia coli* (ETEC), nonpathogenic *E. coli*, and *E. coli* endotoxin. Apical infection of polarized IPEC-J2 monolayers caused a time-dependent decrease in transepithelial electrical resistance (TEER). Microarray analysis showed up-regulation of interleukins when IPEC-J2 were cocultured with *E. coli* strains this has so far never been measured in this cell line. Highest IL8 expression was found with the ETEC strain possessing the F4 fimbrium, suggesting IPEC-J2 cells to be F4 receptor positive, confirmed in a brush border membrane adhesion assay. It is concluded that the innate immune responses to pathogens and LPS makes the IPEC-J2 cell line a suitable model for research on intestinal host pathogen interaction.

## 1. Introduction

The pig's organ sizes, its anatomy, and physiology make it an ideal comparative human model for normal physiology as well as disease research. Further, pigs are omnivores, and the physiology of porcine digestive system is similar to that of humans, making the pig a good model for human intestinal research. For *in vitro* research, hitherto, only cell lines of human origin were used, making a direct comparison quite difficult. Caco2, a human colon cell line, is frequently used because of its great morphological, ultrastructural, and biochemical similarity with small intestinal epithelial cells [1–3], although questions remain about the functional resemblance. The porcine intestinal cell line IPEC-J2 [4, 5] can be an appropriate model through the advantage of direct comparison with the experimental animal and might serve as a good model for humans. Although IPEC-J2, already extensively morphological characterized [5], characterization at the transcriptional level remains limited. 

The human intestinal cell line Caco2 produces interleukins such as IL1, IL6 [6], IL8 [7], and TNF-*α* [8] after inflammatory stimulation by bacteria and metabolites. IL8 levels in the lumen and in the mucosa are elevated during intestinal inflammation states, such as ulcerative colitis and Crohn's disease [9]. Whereas intestinal cell lines seems to be able to mount similar responses as seen *in vivo*, concerns remain on the true competence of cell lines since they may be deficient in certain respects, through, for instance, chromosomal losses. An example of the latter may be the fact that the Caco2 cell line does not express I-FABP [10], an important *in vivo* marker for small intestinal epithelium [11, 12]. 

The central objective of this study was to characterize the transcriptional response of the IPEC-J2 cell line to *E. coli* and LPS from *E. coli*. IPEC-J2 was grown on permeable filter supports as a fully differentiated confluent monolayer as established by transepithelial electrical resistance (TEER) measurement. The IPEC-J2 transcriptional response was analyzed by microarray, using three different stimuli: (1) two pathogens (ETEC), expressing the adhesion factor F4 or not, (2) a nonpathogenic *E. coli* strain (3), and LPS from *E. coli*. The differential expression of pooled samples was determined by microarray analysis at 4 h post-incubation and a selection of genes was validated by qRT-PCR of RNA samples from individual wells to obtain information about variation.

## 2. Materials and Methods

### 2.1. Culture of Epithelial Cells

The porcine jejunal intestinal cell line IPEC-J2 is a nontransformed cell line originating from a neonatal, unsuckled piglet [4]. The culture medium consists of 1 : 1 DMEM (Dulbecco's Modified Eagle Medium)/Ham's F-12 mixture (Invitrogen, Belgium) supplemented with 0.12% sodium bicarbonate (Sigma-Aldrich, Belgium), 15 mM HEPES (Invitrogen, Belgium), 0.5 mM sodium pyruvate (Invitrogen, Belgium), 5% heat-inactivated fetal bovine serum (FBS) (Biochrom AG, International Medical, Belgium), and 1% antibiotic-antimycotic mixture (Sigma-Aldrich, Belgium). All cells were maintained at 37°C in a humidified atmosphere of 5% CO_2_; every other day culture medium was replaced by fresh one, and cells were passaged when they reached confluence. For the microarray experimentations, IPEC-J2 cells between passages 85–92 were seeded onto the Transwell-COL Collagen-Coated Membrane (12-wells) (Corning B.V., The Netherlands) at a high density of 12 × 10^5^ cells/ml (0.5 ml per well as by the manufacturers instructions) to saturate the available area for attachment, hereby avoiding the need for cell division. Cells were allowed to adhere for 48 h before media was replaced with fresh one every other day for 9 days, optimal culturing period as determined in a previous study (to be published), to allow for confluence and tight junction formation. Average cell density in this *in vitro* system was 7 × 10^5^ cells per well and TEER values typically around >3 kΩ cm² at day 9, which indicates the formation of a confluent monolayer with tight junctions. Trans epithelial electrical resistance (TEER) values were measured using an EVOM epithelial Voltohmmeter with STX2 electrodes (World Precision Instruments, USA), and expressed as kΩ cm², the number of repeats ranges from 10–39. Per plate one stimuli was tested of which one well served as a control; in this no cells were present whereby contamination can be checked and served as the control in our calculation for the TEER value. At 4 h post-incubation three replicate plates were used for the pool.

### 2.2. Exposure of IPEC-J2 Monolayer to Inflammatory Stimuli

Prior to coincubation, ±16 h, the IPEC-J2 monolayers (at day 8) were washed twice with phosphate-buffered saline (PBS) and cultured with experimental media without serum and antibiotics. Although serum contains specific proteins for LPS binding, the plasma LPS-binding protein (LBP) and cell membrane CD14 [13], it was chosen to be omitted from the media because it would cause overgrowth of bacteria resulting in too high toxic effects and subsequent cell death. The absence of serum at the luminal side *in vivo* justifies herewith our decision. Treatments included control (uninfected cells), stimulation with 1 *μ*g/ml LPS [14] from *E. coli* O55B5 (L2637, Sigma-Aldrich), CVI-444 (nonpathogenic, F1 positive and without any toxins producing), CVI-1000 *E. coli* O149K91 (F4 (K88ac), LT+, STb+), and CVI-1048 (LT+, STb+) which is identical to CVI-1000 except no F4 [15] (Table 1). The strains were grown from stock overnight (±16 h) at 37°C in LB broth on a rotary shaker (230 rpm), washed three times with PBS (pH 7.2) and resuspended in experimental media at the desired concentration. In the current study, a multiplicity of infection of 1 bacteria to 10 IPEC-J2 cells was used. Higher ratio's (10 : 1 and 1 : 1) resulted in total destruction of cell cultures within 4 h (results not shown). Before RNA isolation IPEC-J2 monolayers were washed three times with PBS (37°C). Samples for the four different inflammatory stimuli were taken after 4 h post-incubation and the control samples at 0 h.

### 2.3. Isolation of RNA

Briefly, total RNA of IPEC-J2 was extracted using TRIzol Reagent (Invitrogen, Belgium) and further purified using a RNeasy Mini Kit and QIAshredder (QIAGEN Benelux, Netherlands). In combination a DNase treatment (RNase-free DNase set, QIAGEN Benelux, Netherlands) was performed to eliminate potential genomic DNA contamination. Microarray analysis of the RNA samples requires specific concentrations: RNA 260/280 ratio between 1.8–2.1 and 260/230 ratio between 1.5–2.0. Pools for microarray analysis were prepared for each group, to obtain sufficient material, by mixing equal amounts of isolated RNA, 0.5 *μ*g/sample. Per microarray 5 *μ*g RNA was required, per well concentration obtained ranged from 3–9 *μ*g.

### 2.4. Microarray Analysis

The Porcine Genome Array (Affymetrix) was used containing 23,937 probe sets to interrogate 23,256 transcripts in pig, which represents 20,201 *Sus scrofa* genes. RNA concentration and purity were determined spectrophotometrically using the Nanodrop ND-1000 (Nanodrop Technologies) and RNA integrity was assessed using a Bioanalyser 2100 (Agilent). Per sample, an amount of 2 *μ*g of total RNA spiked with bacterial RNA transcript positive controls (Affymetrix) was converted to double stranded cDNA in a reverse transcription reaction. Subsequently, the sample was converted and amplified to antisense cRNA and labeled with biotin in an *in vitro* transcription reaction. All steps were carried out according to the manufacturers protocol (Affymetrix). All amplification and labeling reactions were performed on a Biomek 3000 ArrayPlex Workstation (Beckman Coulter). A mixture of purified and fragmented biotinylated cRNA and hybridisation controls (Affymetrix) was hybridised on Affymetrix GeneChip Porcine Genome Arrays followed by staining and washing in a GeneChip fluidics station 450 (Affymetrix) according to the manufacturers procedures. To assess the raw probe signal intensities, chips were scanned using a GeneChip scanner 3000 (Affymetrix).

#### 2.4.1. Analysis of Microarray Data

R (version 2.7.0), a free software environment for statistical computing and graphics, was used in combination with the the *affy* library (version 1.16.0) of BioConductor (http://www.bioconductor.org/) to calculate the MAS 5.0 detection calls and the RMA [16] expression values. The MAS 5.0 detection calls were used to decide whether a signal was significantly above background. To identify differentially expressed genes between two conditions, firstly, all probes with an absent call (A) in both conditions were removed. Subsequently, all probes were removed which had an absolute log2 fold change smaller than 1. To annotate the probes, the latest probe annotations (NetAffx annotation date 2008-12-01 and build 27) were downloaded from the Affymetrix website.

### 2.5. Quantitative Real-Time PCR Analysis

One microgram of total RNA of all individual samples to be analyzed were reverse transcribed utilizing random primers (Promega, Belgium) and dNTP mix (mix of dATP, dCTP, dGTP and dTTP) (VWR, Belgium) for denaturation and Avian Myeloblastosis Virus Reverse Transcriptase (AMV-RTase) with supplemented buffer (Promega, Belgium) and Recombinant RNasin Ribonuclease Inhibitors (Promega, Belgium) for transcription utilizing a standard protocol [17], and subsequently diluted with nuclease-free water (Qiagen) to 10 ng/*μ*l cDNA. 

Primers (Table 2) were designed using Primer3 Output and DNAMAN. For normalization, two housekeeping genes, RPL4 and YWHAZ, were selected from five different candidate housekeeping genes (RPL4, YWHAZ, GAPDH, ACTB and B2M) according to the method of Vandesompele [18].

qRT-PCR on individual samples was performed to confirm differences in mRNA levels as detected by microarray analysis. Per condition six samples were analyzed in triplicate. The method used for the relative quantification of real-time data is the standard curve method. RT-PCR was performed using the ABI Prism 7700 sequence (Applied Biosystems) detection system. In brief, final concentrations used in each 20 *μ*l reaction mix were 10 ng of template cDNA, primers, SYBR Green PCR Master Mix (Applied Biosystems) and RNase-free water (Qiagen, Belgium). Thermal cycling conditions were 95°C for 10 min followed by 40 cycles of 95°C for 15 s and 60°C for 1 min. Negative controls without cDNA template were run with every assay. Products were checked on agarose gels. A standard curve for all genes, including reference genes, was generated using serial dilutions of a pooled sample (cDNA form all conditions). PCR efficiency of 90–110% (3.2 < slope > 3.8) together with a correlation coefficient of >0.99 were accepted.

### 2.6. Statistical Analysis of qRT-PCR Data

Data were analyzed with a univariate General Linear Model that included time as fixed factor. When this variable was significant, factor level means were compared with the Tukey comparison of mean test. *P*-values <  .05 were considered as significant. The statistical analyses were done using SAS, version 9.1.3 for Windows.

### 2.7. Quantification of Intestinal Fatty Acid Binding Protein (I-FABP)

In order to confirm the absence or presence of I-FABP production in the IPEC-J2 cell line, cell extracts and culture supernatants were assayed for I-FABP using the human I-FABP ELISA (Hycult biotech., the Netherlands) as described before [11]. The T84 colonic human carcinoma cell line which produces I-FABP (to be published) was used as a positive control. The negative control for this quantification was the COS-7 cell line, an African green monkey kidney fibroblast-like cell line not of gastro-intestinal origin. The T84 cell line was cultured in 1 : 1 DMEM/Ham's F-12 mixture supplemented with 5% heat-inactivated FBS, with 0.12% sodium bicarbonate, 15 mM HEPES and 0.5 mM sodium pyruvate. The COS-7 cell line is cultured in DMEM supplemented with 10% heat-inactivated FBS and 1% gentamycin. T84 and COS-7 cell lines were grown in multidish 6 well plates (Nunc) at a concentration of 8 × 10^5^ cells/well. IPEC-J2 cells were grown on 6 well Anopore Membranes (0.2 *μ*m, Nunc) (8 × 10^5^ cells/well) essentially as described above. Since expression may be dependent on the stage of differentiation, samples (2 wells per time point) were taken at regular time points over a period of 14 days. Cells were finally diluted in 500 *μ*l water and the final concentration being used in the ELISA was 4 × 10^4^cells/test.

### 2.8. Bacterial Cell Adhesion Assay

IPEC-J2 fully differentiated cell monolayers were washed with sterile PBS and detached through the use of a scraper. Cells were pelleted and resuspended in PBS at a concentration of 1 × 10^6^ cells/ml, in the presence of 1% D-mannose to prevent binding by type I fimbriae if present. Bacterial cell adhesion assay was performed essentially as described before [19], using isolated brush borders from a F4 receptor positive (F4R+) pig as control. Briefly, to 0.5 ml cell suspension (IPEC-J2 or brush borders), 0.5 ml of a suspension containing 10^8^ bacteria/ml PBS (2% D-mannose) was added and the sample was gently mixed on a shaker at room temperature for 30 min. A small aliquot was put on a slide under a cover slip, and bacterial adherence was determined by phase contrast microscopy (magnification, x400). The number of bacteria attached to IPEC-J2 cells or well-defined brush borders (*n* = 5–20) were counted. Samples with 0-2 bacteria/cell or brush border were considered F4R-, samples exceeding this are judged F4 receptor positive (F4R+).

## 3. Results and Discussion

### 3.1. Transepithelial Electrical Resistance (TEER) Measurements (Table 3)

IPEC-J2 monolayers with a TEER value of >3 kΩ cm² were used for all experimentations. Cells were grown to full differentiation as evidenced by TEER, and subsequently, different inflammatory stimuli were applied. A 6% decline is caused through the handlings the TEER measurements require, as seen for the control. Coincubation with LPS (1 *μ*g/ml), caused a 22% decline after 4 h of exposure. Coincubation with *E. coli* strains resulted in different degrees of reduction probably related to differences in virulence. The nonpathogenic strain expressing F1, did reduce TEER to 63%. LPS, present in all *E. coli* preparations, is most likely responsible for at least part of the reduction of TEER by the nonpathogenic strain. Other causes for the reduction in TEER with life strains could be bacterial metabolites. A large significant percentual decline of TEER values after 4 h coincubation was seen with the two ETEC strains CVI-1000 and CVI-1048. The largest reduction in TEER was caused by CVI-1000 which expresses the F4 fimbrium. This is consistent with the role of F4 as an important virulence factor and it also suggests that IPEC-J2 expresses the F4 receptor. Furthermore, it is concluded that differences in TEER reduction between the different *E. coli* strains are related to differences in virulence. At 8 h post-incubation, no pure and uncontaminated RNA could be isolated from both ETEC infected cultures (data not shown), which is consistent with eukaryotic cell destruction.

### 3.2. Microarray Analysis

#### 3.2.1. Summary of Microarray Analysis of Cocultures versus Control (4 h versus 0 h)

The gene expression patterns of IPEC-J2 cells cocultured for 4 h were compared with IPEC-J2 cells at 0 h hereby revealing a large number of genes to be regulated in LPS (175), CVI-444 (27), CVI-1000 (58), and CVI-1048 (166). The differences between the number of genes regulated in the *E. coli* strains may be related to different pathogenicity or and hence may differ in the magnitude of stimulation.

 Genes that all four coculturing experiments have in common are TXNIP, PEG10, *S. scrofa* 28S rRNA and three transcribed loci. TNXIP and also TRX (see Supplementary Material available on line at doi:10.1155/2011/469583) regulate the cellular redox balance, promotion of cell growth, inhibition of apoptosis, and modulation of inflammation [20]. PEG10 has a known function in inhibiting the TGF-beta signaling pathway.

 A wide array of genes was found to be differentially regulated. Among those, many transcribed loci and a variety of genes which relationship to inflammatory stimuli is unclear. Therefore, we mainly limited the description of the microarray results to the annotated immune genes.

Genes that are found in three out of four treatments include several with a specific function in the immune and inflammatory response, CXCL2, IL8, AREG and CYP1A1 [21]. Herewith, AREG promotes the growth of normal epithelial cells and inhibits the growth of certain aggressive carcinoma cell lines.

Genes in common for two out of four treatments include genes such as FBXO32, IRG6, OLR1, MX1, DDX58, A2M, SERPINA1, IL1A classified as having cytokine activity and involved in the immune response and chemokine signalling pathway.

Most interesting, the expression of genes involved in the innate immune response were influenced, such as IL-1*α*, IL8, chemokine (C-X-C motif) ligand 2, IRG6, ceruloplasmin, CYP1A1, A2M, SERPINA1, MMP-13, FBXO32, MX1, DDX58, TXNIP and TRXR1 (Table 4 and see Supplementary Material). Ceruloplasmin and A2M are acute phase reactants. SERPINA is a serum protease inhibitor, a cytokine carrier, and involved in host defence like A2M [22]. The JNK-MMP13 signaling pathway plays an essential role in regulating the innate immune cell migration in response to severe injury *in vivo* [23]. FBXO32 is involved in the Ubl conjugation pathway, which has a central role in the activation of inflammation [24]. From the genes found here, several have been described in human epithelial cell lines too such as IL8 (for Caco2 [25, 26], for T84 and HT-29 [27]), A2M (for Caco2, [28]), a SERPINA gene (SERPINA3) in Caco2 cells exposed to cholera toxin [29] and CYP1A1 (for Caco2 [30]). *In vivo,* increased IL8 levels appeared to play an important role in infection resolution of ETEC [31]. *Salmonella* infections in pigs induced the production of IL8 [32–34]. SERPINA3, IL-1*β*, chemokine (C-X-C motif) ligand 13, MMP1 and MMP3 expression was found in duodenal mucosa during acute cholera [29]. Almost all genes found here have also been found in human and in cell lines in inflammatory conditions. The only exception is IRG6, which is apparently unique to pigs.

 As stated earlier, the pooled approach has limitations, in terms of quantification, and especially variation herefore a validation of microarray results on individual samples needs to be performed.

#### 3.2.2. Validation of Selected Genes with qRT-PCR (Figure 2)

**Figure 1:**
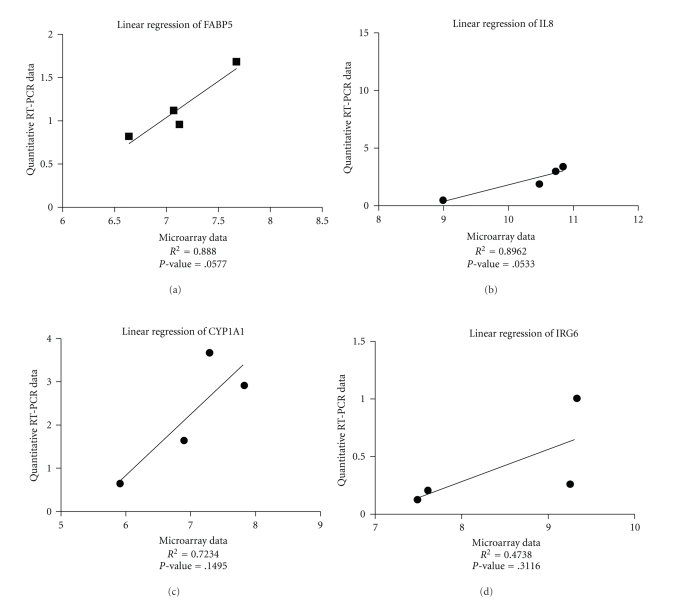
Linear regression of qRT-PCR data versus microarray data of IL8, IRG6, CYP1A1 and FABP5. The goodness of fit (*r* ^2^) and *P*-value are given.

**Figure 2:**
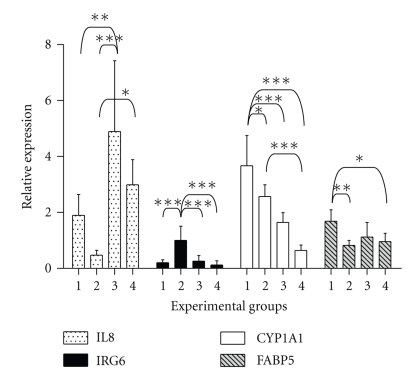
qRT-PCR analysis of IL8, IRG6, CYP1A1 and FABP5, in response to the four treatments at 4 h. LPS (1), CVI-444 (2), CVI-1000 (3) and CVI-1048 (4) (mean with S.D. of *n* = 5-6). Asterisks indicate significant differences between treatments. *.05 < *P* < .01, **.01 < *P* < .005, ***P < .005.

Microarray analysis was validated through quantifying the expression with RT-PCR on seven selected genes, namely cytochrome P450 1A1 (CYP1A1), interleukin 8 (IL8), inflammatory response protein 6 (IRG6), epidermal-fatty acid binding protein (FABP5), liver-FABP (FABP1), intestinal-FABP (FABP2 or I-FABP) and pancreatitis-associated protein (PAP). The genes CYP1A1, IL8, IRG6 and FABP5 were chosen for their present call in the MAS 5.0 detection calls and for their possible involvement in the immune response. For those genes the qRT-PCR data correlated well with the microarray data as seen in their linear regression analysis (Figure 1). Figure 2 displays the relative expression of the four genes we were able to quantify with RT-PCR. The first gene of which the expression was determined in the four experimental groups was IL8, a potent neutrophil and T-lymphocyte chemoattractant. Analysis shows that IPEC-J2 cells infected with the ETEC strain CVI-1000 has the highest relative expression, significantly different from the treatment with LPS and CVI-444, which agrees with the microarray data (Table 4). In absolute values the difference in response of IPEC-J2 to CVI-1000 and CVI-1048 is smaller in our microarray data than found with qRT-PCR. This could result from the pooled approach of the microarray experiment. However, where previous results demonstrated the lack of response of IPEC-J2 to LPS in terms of IL8 mRNA expression due to hyporesponsiveness [35] this is not consistent with our results presumably due to the 10-fold higher concentration used. In other studies expression of IL8 is demonstrated for intestinal human cell lines, for example, SW620 and HT29 [36]. Analysis of the expression pattern of IRG6 in the different treatments revealed a significant upregulation of this gene in IPEC-J2 infected with the *E. coli* strain CVI-444. Interestingly, the relative expression of IRG6 seems to be inversely related to that of IL8, which could suggest a possible role of IRG6 in the downregulation of inflammation. CYP1A1 was significantly upregulated as determined by qRT-PCR in the treatment with LPS against all other coculture experiments, and a similar upregulation was found in the microarray analysis (Table 4). FABP5 was upregulated a 1.06 times a 2 fold induction in the microarray analysis after stimulation by LPS (see Supplementary Material), which is in absolute values not that different from qRT-PCR data (Figure 2). 

The genes FABP1, FABP2 and PAP were absent in the MAS 5.0 detection calls, and the absence of expression was confirmed by qRT-PCR. PAP is in the intestine believed to be specific for Paneth-cells [37], and is not expected to be expressed here. In contrast, FABP1 and FABP2 are markers for small intestinal epithelium. Furthermore, for FABP2 (I-FABP) also no protein was detected. Whatever the reasons may be for nonexpression, it is concluded that the qRT-PCR data confirm the microarray analysis in the case of absent calls.

### 3.3. Intestinal Fatty Acid Binding Protein (I-FABP) Production

**Figure 3:**
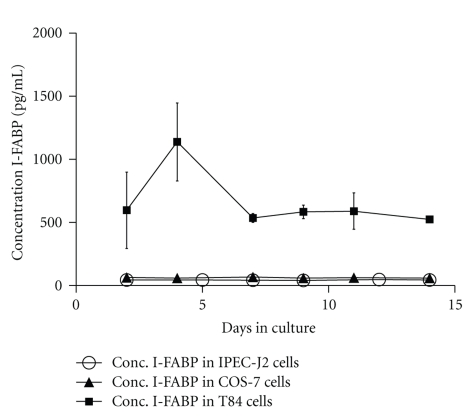
Intracellular concentration of I-FABP (mean ± S.D. of *n* = 6–8) in the three different cell lines during 14 days of culture (open circle: IPEC-J2 cells, closed triangle: COS-7 cells and closed rectangle: T84 cells).

No I-FABP could be detected in the IPEC-J2 cell line by ELISA, nor in the negative control cell line COS-7. In contrast, I-FABP was clearly demonstrated in T84 cells as expected (Figure 3). 

Both microarray and ELISA results suggest that IPEC-J2 does not express I-FABP, and is in that respect similar to human intestinal epithelial cell lines such as Caco2 [10] and HT-29 [38]. This could be caused by that expression of I-FABP in IPEC-J2 requires different growth factors, and hormones, or that IPEC-J2 simply misses the gene coding for this protein.

### 3.4. Bacterial Cell Adhesion

The bacterial cell adhesion assay with CVI-444, CVI-1000 and CVI-1048 revealed that CVI-1000 binds in a non-mannose sensitive manner to IPEC-J2 as well as to the F4R+ brush border membranes, which was not the case for CVI-1048. CVI-444 shows no binding which is consistent with the presence of a mannose sensitive F1 receptor (Table 5). This indicates the presence of a F4 receptor on IPEC-J2 cells on all time points. It is known that not all pigs express the F4 receptor, it is a Mendelian trait, and the exact gene responsible is hitherto unknown [39]. The results of the brush border adhesion assay indeed suggest that IPEC-J2 does express the F4 receptor, which makes it a suitable model for research into host pathogen interactions.

## 4. Conclusions

The purpose of our study was to further explore the transcriptional responses of the IPEC-J2 cell line to an *E. coli* strain, two ETEC strains and LPS from *E. coli*. *In vivo*, porcine intestinal epithelial cells secrete cytokines in response to inflammatory or pathogenic stimuli [19], and human cell lines such as Caco2 or other epithelial cell lines [8, 26, 27] appear to have similar properties *in vitro*. Previous results on IPEC-J2 demonstrate that these cells can produce some cytokine and chemokine mediators as a response to bacterial invasion [35]. Hitherto, no such microarray data are available on IPEC-J2 cells. 

 Our microarray results show that IPEC-J2 cells are capable of expressing a host of genes in response to inflammatory stimuli. The changes found although small can still be relevant because they are an integral part of the physiological response and are essential for the identification of the physiological processes that are affected by the challenge [40]. Noteworthy is the fact that not all genes react in a similar way, for example, up or downregulated, to the three *E. coli* strains and purified LPS being used. This involves only three genes, A2M, SERPINA1 and a similar gene to NP_001068.1 UDP glycosyltransferase 2 family (Table 4 and see Supplementary Material). An explanation for this phenomenon is hard to offer, but since it applies only to a small minority of genes, it is unlikely to be very significant.

 Furthermore, the qRT-PCR data of selected genes largely confirmed the microarray results. In case of the genes which showed differential expression in the microarray analysis, a good correlation was found between the expression as established by microarray data and qRT-PCR data, with the exception of FABP5. What is immediately evident from the qRT-PCR data is that there is no similarity between the reaction patterns of all four genes to the different stimuli. For instance, whereas CYP1A1 is significantly higher expressed in response to LPS, this is clearly not the case for IL8. Interestingly, only the expression of IL8 correlates (positively) with the TEER values. IL8 also appears to relate positively with increasing virulence, the highest level found with the F4 positive ETEC strain. This suggests that IL8 levels, at the transcriptional level, are directly related to the intensity of the stimulus. This is consistent with the fact that IL6 and IL8 levels in the lumen and in inflamed mucosa are related to severity of disease in ulcerative colitis and Crohn's disease [6, 9]. 

To conclude, it appears that the intestinal epithelial cells IPEC-J2 grown as a monolayer with functional differentiated cells is an appropriate *in vitro* model system as shown by transcriptional analysis. They are capable to produce pro-inflammatory mediators as a defense against pathogens, such as interleukins and chemokines, which mimics the reaction observed *in vivo* and exhibit features similar to that of human intestinal cell lines such as Caco2. Furthermore, the fact that IPEC-J2 cells appear to express a functional F4 receptor makes this cell line particularly suited for the study of ETEC host interactions.

##  Conflict of Interests

All authors declare no conflict of interests.

## Supplementary Material

Complete list of differentially expressed genes (additional to Table 4).

## Figures and Tables

**Table 1:** Different inflammatory stimuli with their specifications and concentrations used.

	LPS	Enterotoxins LT and STb	Fimbrium	Concentration
Lipopolysaccharide	+			1 *μ*g/ml
CVI-444	+		F1	MOI = 1 : 10
CVI-1000	+	+	F4	MOI = 1 : 10
CVI-1048	+	+		MOI = 1 : 10

**Table 2:** Primer sequences used in this study.

Symbol	Name	Probe set ID	Forward primer	Reverse primer
RPL4	Ribosomal protein L4	Ssc.12277.1.S1_at	GAGAAACCGTCGCCGAAT	GCCCACCAGGAGCAAGTT
YWHAZ	Tyrosine 3-monooxygenase/tryptophan 5-monooxygenase activation protein, zeta polypeptide	Ssc.9681.1.A1_at	AGCAGATGGCTCGAGAAT	GCAACCTCAGCCAAGTAAC
GAPDH	Glyceraldehyde-3-phosphate dehydrogenase	Ssc.14942.1.S1_at	GGTCGGAGTGAACGGATTTG	ACTGTGCCGTGGAATTTGC
ACTB	Beta actin	Ssc.13874.1.S1_at	CTACGTCGCCCTGGACTTC	GATGCCGCAGGATTCCAT
B2M	Beta-2-microglobulin	Ssc.12348.1.S1_at	TCGGGCTGCTCTCACTGT	GACTGCTCCGCGTTCATC
IL8	Interleukin-8	Ssc.658.1.S1_at	TCACAAGCTCCTAGGACCAGA	CAGAACTGCAGCCTCACAGA
PAP	Pancreatitis-associated protein	Ssc.16470.1.S1_at	GAAGATTCCCCAGCAGACAC	AGGACACGAAGGATGCCTC
FABP1	Liver fatty acid binding protein	Ssc.604.1.S1_at	CCAAGTACAGAGCCAGGAAAA	CCCGGTAGTGATGGTCAACT
FABP2	Intestinal fatty acid binding protein	Ssc.16525.1.S1_at	TGAATCAGCTGGAGACTATGG	TTTACCACGTTAATACCCATTTTT
FABP5	Epidermal fatty acid binding protein	Ssc.5549.1.S1_at	CCAGGCTCTAGGCACCAGT	GGCCATTCCCACTCCTACTT
IRG6	Inflammatory response protein 6	Ssc.286.1.S1_s_at	CATCAATCGCTTCAATGTGG	ACCAAGCAGGACACGTCTTT
CYP1A1	Cytochrome P450 1A1	Ssc.208.1.S1_at	CAACACGTCCCTGGATCTCT	ATCCGACAGCTGGATATTGG

**Table 3:** Development of the transepithelial electrical resistance (TEER, in kΩ cm^2^ as average (±S.D.)) in time. Letters indicate significant differences in the same row. Last column shows final TEER values as a percentage of 0 h.

	0 h	2 h	4 h	4 h versus 0 h (%)
control	6.11 ± 1.17^(a)^	5.64 ± 1.13^(b)^	5.73 ± 1.03^(b)^	94
LPS	3.50 ± 0.84^(a)^	2.62 ± 0.80^(b)^	2.74 ± 0.99^(b)^	78
CVI-444	4.62 ± 1.30^(a)^	3.38 ± 1.07^(b)^	2.90 ± 0.92^(b)^	63
CVI-1000	4.85 ± 1.24^(a)^	4.01 ± 0.98^(b)^	0.10 ± 0.15^(c)^	2
CVI-1048	4.84 ± 1.04^(a)^	3.76 ± 0.79^(b)^	1.29 ± 0.65^(c)^	27

**Table 4:** Microarray data expressed as a log2 fold change of the transcriptional response of IPEC-J2 cocultured with inflammatory stimuli as compared to the control. Genes common to at least two out of four treatments are given (for full list see Supplementary Material).

	log2 fold change							
Probeset ID	LPS versus control	CVI-444 versus control	CVI-1000 versus control	CVI-1048 versus control	Public ID	Gene title	Gene symbol	Tentative function (UniProtKB)
Ssc.19163.1.S1_at	−1,51	−1,42	−1,10	−1,71	BF078930	Transcribed locus	—	
Ssc.19268.2.A1_at	−1,09	−1,09	−1,11	−1,16	CF359637	Transcribed locus, moderately similar to NP_660274.1 chromosome 14 open reading frame 143 (*H. sapiens*)	—	Calcium ion binding
AFFX-SSC-28SrRNA_at	−1,16	−1,46	−1,26	−1,20	AFFX-SSC-28SrRNA	*S. scrofa* 28S rRNA	—	
Ssc.24007.1.S1_at	2,16	1,04	1,38	1,68	CK450048	Paternally expressed 10	PEG10	Nucleic acid binding
Ssc.15937.1.A1_at	1,50	1,54	1,41	1,62	AF248308.1	Transcribed locus, moderately similar to XP_375341.1 similar to Ig heavy chain - human (fragment) (*H. sapiens*)	—	
Ssc.19692.1.S1_at	1,34		1,87	1,92	BF078671	Chemokine (C-X-C motif) ligand 2	CXCL2	Immune response
Ssc.4871.1.S1_at	1,85		2,21	2,57	NM_001001861.1	Chemokine (C-X-C motif) ligand 2	CXCL2	Immune response
Ssc.658.1.S1_at	1,93		2,29	2,18	NM_213867.1	Interleukin 8	IL8	Cytokine, inflammatory response, immune response
Ssc.14467.2.S1_a_at	1,99	1,24	1,14		AY028311.1	Amphiregulin	AREG	Cytokine, growth factor
Ssc.286.1.S1_s_at	−1,72			−1,84	NM_213817.1	Inflammatory response protein 6	IRG6	Antiviral defense, defense response to virus
Ssc.208.1.S1_at	2,22	2,75	1,83		NM_214412.1	Cytochrome P450 1A1	CYP1A1	Oxidation reduction
Ssc.113.1.S2_at			1,12	2,16	NM_214029.1	Interleukin 1, alpha	IL1A	Cytokine, inflammatory response, immune response
Ssc.113.1.S1_at			1,28	2,58	M86730.1	Interleukin 1, alpha	IL1A	Cytokine, inflammatory response, immune response
Ssc.10453.1.S1_at			−1,07	−1,42	BF713714	Transcribed locus, highly similar to NP_000087.1 ceruloplasmin (ferroxidase) (*H. sapiens*)	—	Oxidoreductase, transport
Ssc.26317.1.S1_at	1,23			−1,55	AY509877.1	Alpha-2-macroglobulin	A2M	Endopeptidase inhibitor activity
Ssc.7090.1.A1_at	1,46			−1,48	NM_214395.1	Serpin peptidase inhibitor, clade A (alpha-1 antiproteinase, antitrypsin), member 1	SERPINA1	Protease inhibitor, serine protease inhibitor
Ssc.16053.1.S1_at				−1,08	AF069643.1	Matrix metalloproteinase 13 precursor	MMP-13	Hydrolase, protease, metalloprotease
Ssc.4368.3.S1_at	−1,73			−1,19	BP463181	F-box protein 32	FBXO32	Ubl conjugation pathway
Ssc.4368.1.S1_at	−1,59			−1,32	BI817204	F-box protein 32	FBXO32	Ubl conjugation pathway
Ssc.221.1.S1_at	−1,09			−1,55	NM_214061.1	Myxovirus (influenza virus) resistance 1, interferon-inducible protein p78	MX1	Antiviral defense, reponse to virus
Ssc.21.1.S1_s_at	−1,05			−1,66	AF319661.1	DEAD (Asp-Glu-Ala-Asp) box polypeptide 58	DDX58	Antiviral defense, immune response, innate immunity
Ssc.15888.1.S1_at	−1,22			−1,07	NM_213805.1	Oxidized low density lipoprotein (lectin-like) receptor 1	OLR1	Cell adhesion, immune response, inflammatory response
Ssc.16648.1.S1_at	−1,28	−1,35	−2,30	−3,34	CK458095	Thioredoxin interacting protein	TXNIP	Cell cycle, transcription, transcription regulation

**Table 5:** Bacterial adhesion assay in the presence of 1% D-mannose. Numbers are the average (±S.D.) of adherent bacteria per cell or brush border (N.A. = not applicable).

*E. coli *strains	F4R+ brush border membranes	IPEC-J2 14d culture flask	IPEC-J2 10d membrane support	IPEC-J2 15d membrane support	IPEC-J2 21d membrane support
CVI-444 (F1+)	N.A.	0.31 ± 0.74	0.58 ± 1.16	0.29 ± 0.49	0.30 ± 0.47
CVI-1000 (F4+)	2.17 ± 1.88	9.17 ± 7.20	2.82 ± 4.26	7.43 ± 5.72	7.59 ± 5.47
CVI-1048 (F4-)	0.00 ± 0.00	0.63 ± 1.01	0.62 ± 0.77	0.67 ± 0.98	0.86 ± 0.90
