# Supplementary material for: Preliminary Characterization of the Transcriptional Response of the Porcine Intestinal Cell Line IPEC-J2 to Enterotoxigenic Escherichia coli, Escherichia coli, and E. coli Lipopolysaccharide
Source: Comp Funct Genomics. 2010 Dec 29;2010:469583. doi: 10.1155/2010/469583 (PMC3034941; doi:10.1155/2010/469583)
Supplement: Supplementary file 1 — Complete list of differentially expressed genes (additional to Table 4). [file 469583.f1.pdf]

| ProbesetID | log2 fold-change<br>LPS vs control | E. coli 444 vs control | E. coli 1000 vs control | E. coli 1048 vs control | Public ID | Gene Title | Gene Symbol | Tentative function (UniProtKB) |
|---|---|---|---|---|---|---|---|---|
| Ssc.19163.1.S1_at | -1.51 | -1.42 | -1.10 | -1.71 | BF078930 | Transcribed locus | --- | |
| Ssc.19268.2.A1_at | -1.09 | -1.09 | -1.11 | -1.16 | CF359637 | Transcribed locus, moderately similar to NP_660274.1 chromosome 14 open reading frame 143 (Homo sapiens) | --- | Calcium ion binding |
| AFFX-SSC-28SrRNA_at | -1.16 | -1.46 | -1.26 | -1.20 | AFFX-SSC-28SrRNA | Sus scrofa 28S rRNA | --- | |
| Ssc.24007.1.S1_at | 2.16 | 1.04 | 1.38 | 1.68 | CK450048 | paternally expressed 10 | PEG10 | Nucleic acid binding |
| Ssc.15937.1.A1_at | 1.50 | 1.54 | 1.41 | 1.62 | AF248308.1 | Transcribed locus, moderately similar to XP_375341.1 similar to Ig heavy chain - human (fragment) (Homo sapiens) | --- | |
| Ssc.19692.1.S1_at | 1.34 | | 1.87 | 1.92 | BF078671 | chemokine (C-X-C motif) ligand 2 | CXCL2 | Immune response |
| Ssc.4871.1.S1_at | 1.85 | | 2.21 | 2.57 | NM_001001861.1 | chemokine (C-X-C motif) ligand 2 | CXCL2 | Immune response |
| Ssc.658.1.S1_at | 1.93 | | 2.29 | 2.18 | NM_213867.1 | interleukin 8 | IL8 | Cytokine, inflammatory response, immune response |
| Ssc.14467.2.S1_a_at | 1.99 | 1.24 | 1.14 | | AY028311.1 | amphiregulin | AREG | Cytokine, growth factor |
| Ssc.286.1.S1_s_at | -1.72 | | | -1.84 | NM_213817.1 | inflammatory response protein 6 | IRG6 | Antiviral defense, defense response to virus |
| Ssc.208.1.S1_at | 2.22 | 2.75 | 1.83 | | NM_214412.1 | cytochrome P450 1A1 | CYP1A1 | Oxidation reduction |
| Ssc.113.1.S2_at | | | 1.12 | 2.16 | NM_214029.1 | interleukin 1, alpha | IL1A | Cytokine, inflammatory response, immune response |
| Ssc.113.1.S1_at | | | 1.28 | 2.58 | M86730.1 | interleukin 1, alpha | IL1A | Cytokine, inflammatory response, immune response |
| Ssc.10453.1.S1_at | | | -1.07 | -1.42 | BF713714 | Transcribed locus, highly similar to NP_000087.1 ceruloplasmin (ferroxidase) (Homo sapiens) | --- | Oxidoreductase, transport |
| Ssc.26317.1.S1_at | 1.23 | | | -1.55 | AY509877.1 | alpha-2-macroglobulin | A2M | Endopeptidase inhibitor activity |
| Ssc.7090.1.A1_at | 1.46 | | | -1.48 | NM_214395.1 | serpin peptidase inhibitor, clade A (alpha-1 antiproteinase, antitrypsin), member 1 | SERPINA1 | Protease inhibitor, serine protease inhibitor |
| Ssc.16053.1.S1_at | | | | -1.08 | AF069643.1 | matrix metalloproteinase 13 precursor | MMP-13 | Hydrolase, protease, metalloprotease |
| Ssc.4368.3.S1_at | -1.73 | | | -1.19 | BP463181 | F-box protein 32 | FBXO32 | Ubl conjugation pathway |
| Ssc.4368.1.S1_at | -1.59 | | | -1.32 | BI817204 | F-box protein 32 | FBXO32 | Ubl conjugation pathway |
| Ssc.221.1.S1_at | -1.09 | | | -1.55 | NM_214061.1 | myxovirus (influenza virus) resistance 1, interferon-inducible protein p78 | MX1 | Antiviral defense, reponse to virus |
| Ssc.21.1.S1_s_at | -1.05 | | | -1.66 | AF319661.1 | DEAD (Asp-Glu-Ala-Asp) box polypeptide 58 | DDX58 | Antiviral defense, immune response, innate immunity |
| Ssc.15888.1.S1_at | -1.22 | | | -1.07 | NM_213805.1 | oxidized low density lipoprotein (lectin-like) receptor 1 | OLR1 | Cell adhesion, immune response, inflammatory response |
| Ssc.16648.1.S1_at | -1.28 | -1.35 | -2.30 | -3.34 | CK458095 | Thioredoxin interacting protein | TXNIP | Cell cycle, transcription, transcription regulation |

| | | | | | | | | |
|---|---|---|---|---|---|---|---|---|
| Ssc.6097.1.S1_at | 1.16 | | | | CN166758 | Thioredoxin reductase | TRXR1 | Oxidoreductase |
| Ssc.5549.2.S1_at | 1.06 | | | | CB468944 | Fatty acid binding protein 5 | FABP5 | Transport, lipid binding |
| Ssc.16470.1.S1_a_at | | | | | CO994432 | Transcribed locus, moderately similar to NP_002571.1 pancreatitis-associated protein (Homo sapiens) | PAP | Acute phase, inflammatory response |
| Ssc.604.1.S1_at | | | | | BX924285 | Liver fatty acid binding protein | FABP1 | Transport, lipid binding |
| Ssc.16525.1.S1_at | | | | | CB285696 | Transcribed locus, moderately similar to NP_000125.1 fatty acid binding protein 2, intestinal (Homo sapiens) | FABP2 | Transport, lipid binding |
| Ssc.11079.1.A1_at | -1.29 | | -1.04 | -1.58 | NM_213936.1 | ribonuclease, RNase A family, 4 | RNASE4 | Endonuclease, hydrolase, nuclease |
| Ssc.24282.1.S1_at | 2.44 | | 1.89 | 2.55 | AJ747030 | Transcribed locus, moderately similar to NP_002080.1 chemokine (C-X-C motif) ligand 2 (Homo sapiens) | --- | Immune response, inflammatory response |
| Ssc.11879.1.A1_at | 1.37 | | 1.11 | 1.33 | BQ600284 | Transcribed locus, moderately similar to NP_001245.1 CDC6 cell division cycle 6 homolog (S. cerevisiae) (Homo sapiens) | --- | Cell cycle, cell divison, DNA replication, mitosis |
| Ssc.10686.1.S1_at | | -1.13 | -1.10 | -1.56 | AJ656758 | Transcribed locus | --- | |
| Ssc.30532.1.A1_at | -1.88 | | -1.12 | -2.04 | CO993367 | Transcribed locus | --- | |
| Ssc.9238.1.A1_at | -1.44 | -1.08 | | -1.47 | BF709956 | Transcribed locus | --- | |
| Ssc.6788.1.A1_at | -1.16 | | -1.03 | -1.27 | AJ683882 | Transcribed locus | --- | |
| Ssc.22530.1.S1_at | -1.14 | -1.54 | | -1.62 | CF795706 | Transcribed locus | --- | |
| Ssc.6189.1.A1_at | 1.94 | | 1.21 | 1.24 | BQ601447 | Transcribed locus | --- | |
| Ssc.6550.1.A1_at | -1.29 | | -1.04 | -1.56 | CK462428 | Transcribed locus | --- | |
| AFFX-Ss_IRP_3_at | -1.71 | | | -1.80 | AFFX-r2-TagQ-5 | Clone 76H1 | --- | |
| Ssc.1257.3.S1_at | -1.24 | | | -1.30 | BP460358 | similar to phosphoinositide-3-kinase interacting protein 1 | LOC100155147 | Integral to membrane |
| AFFX-Ss_IRP_M_at | -1.14 | | | -1.27 | AFFX-r2-TagQ-5 | Clone 76H1 | --- | |
| Ssc.4679.1.S1_at | -1.10 | | | -1.01 | CN157788 | angiogenin | ANG1 | Angiogenesis, differentiation |
| Ssc.6948.1.A1_at | -1.01 | | | -1.43 | BI404568 | similar to neuroserpin | LOC100154352 | Protease inhibitor, serine protease inhibitor |
| Ssc.1843.1.S1_at | 1.06 | | | 1.06 | BX925328 | similar to v-myb myeloblastosis viral oncogene homolog (avian)-like 2 | LOC100157175 | |
| Ssc.1843.2.S1_at | 1.07 | | | 1.04 | AW785539 | similar to v-myb myeloblastosis viral oncogene homolog (avian)-like 2 | LOC100157175 | |
| Ssc.14243.1.S1_at | 1.19 | | | 1.03 | BX671487 | cyclin B | CCNB1 | Cell cycle, cell division |
| Ssc.2753.1.S1_at | 1.19 | | | 1.04 | CN166341 | Polo-like kinase 1 (Drosophila) | PLK1 | Cell cycle, cell division, mitosis |
| Ssc.4747.1.S1_at | 1.38 | | 1.01 | | NM_001003662.1 | follistatin | FST | |
| Ssc.13476.1.A1_at | 1.73 | | | 1.26 | CN162607 | paternally expressed 10 | PEG10 | Nucleic acid binding |
| AFFX-SSC-5SrRNA_at | | -1.94 | | -1.55 | AFFX-SSC-5SrRNA | | --- | |
| Ssc.24401.1.A1_s_at | | | -1.23 | -1.98 | CK457947 | Low molecular weight kininogen | KNG | Cysteine-type endopeptidase inhibitor activity |
| Ssc.13961.2.A1_at | | | 1.13 | 1.28 | CO994537 | Eukaryotic translation elongation factor 1 alpha 1 | EEF1A1 | Elongation factor |

| | | | | | | | | |
|---|---|---|---|---|---|---|---|---|
| Ssc.22068.1.S1_at | | | 1.87 | 1.49 | CN154316 | Transcribed locus, highly similar to NP_077016.1 hypothetical protein MGC4504 (Homo sapiens) | --- | Apoptosis, unfolded protein response |
| Ssc.10441.1.S1_at | 1.57 | | | -1.01 | CN159120 | Transcribed locus, moderately similar to NP_001068.1 UDP glycosyltransferase 2 family, polypeptide B17 (Homo sapiens) | --- | Glycosyltransferase, transferase |
| Ssc.1977.1.S1_at | -1.03 | -1.29 | | | BF075309 | Transcribed locus, moderately similar to NP_035014.1 N-myc downstream regulated gene 1 (Mus musculus) | --- | Response to metal ion |
| Ssc.3391.3.S1_at | 1.04 | | | 1.29 | BF194088 | Transcribed locus, moderately similar to NP_005429.1 FOS-like antigen 1 (Homo sapiens) | --- | Cellular defense response, response to virus |
| Ssc.3391.1.S1_at | 1.10 | | | 1.33 | BI400819 | Transcribed locus, moderately similar to NP_005429.1 FOS-like antigen 1 (Homo sapiens) | --- | Cellular defense response, response to virus |
| Ssc.2099.1.S1_at | 1.11 | | | 1.06 | CO939739 | Transcribed locus, highly similar to NP_004847.2 kinesin family member 23 (Homo sapiens) | --- | Cell cycle, cell divison, mitosis |
| Ssc.16603.1.A1_at | 1.14 | | | -1.62 | BI181630 | Transcribed locus, moderately similar to NP_000005.1 alpha-2-macroglobulin (Homo sapiens) | --- | Endopeptidase inhibitor activity |
| Ssc.19346.1.S1_at | 1.14 | | | 1.05 | CK451940 | Transcribed locus, moderately similar to NP_071394.2 claspin homolog (Xenopus laevis) (Homo sapiens) | --- | Cell cycle, DNA repair, DNA damage |
| Ssc.19326.1.A1_at | 1.15 | | | 1.21 | CF368489 | Transcribed locus, moderately similar to NP_057427.2 centromere protein F, 350400ka (mitosin) (Homo sapiens) | --- | Developmental protein |
| Ssc.4976.1.S1_at | 1.19 | | | 1.01 | CK464589 | Transcribed locus, moderately similar to NP_006333.1 transforming, acidic coiled-coil containing protein 3 (Homo sapiens) | --- | |
| Ssc.22370.1.S1_at | 1.19 | | | 1.07 | CF792337 | Transcribed locus, highly similar to NP_076999.1 defective in sister chromatid cohesion homolog 1 (S. cerevisiae) (Homo sapiens) | --- | Cell cycle, DNA replication |
| Ssc.27275.1.A1_at | 1.19 | | | 1.09 | CK455395 | Transcribed locus, moderately similar to NP_060286.1 hypothetical protein FLJ20425 (Homo sapiens) | --- | Protein binding |
| Ssc.4430.1.S1_at | 1.05 | | | 1.13 | CF788826 | Transcribed locus, highly similar to NP_079021.2 SHC SH2-domain binding protein 1 (Homo sapiens) | --- | |
| Ssc.21011.1.S1_at | 1.92 | | | 1.05 | Z99081 | Transcribed locus, highly similar to NP_000080.1 collagen, type I, alpha 2 (Homo sapiens) | --- | Rho protein signal transduction |
| Ssc.22564.1.A1_at | | -3.36 | | -2.77 | CF795787 | Transcribed locus, weakly similar to XP_379739.1 LOC401630 (Homo sapiens) | --- | |
| Ssc.26282.1.S1_a_at | | -1.26 | -1.09 | | CF795087 | Transcribed locus, weakly similar to NP_690845.1 Saccharomyces cerevisiae TAR1 gene | --- | Regulation of cellular respiration |
| Ssc.4104.1.S1_at | | 1.18 | | 1.83 | CN162948 | Transcribed locus, highly similar to NP_061931.1 DNA-damage-inducible transcript 4 (Homo sapiens) | --- | Apoptosis, negative regulation of signal transcription |
| Ssc.10471.1.A1_at | | | -1.20 | -1.26 | BF702214 | Transcribed locus, moderately similar to XP_128791.2 THUMP domain containing 2 (Mus musculus) | --- | |
| Ssc.9136.1.S1_at | | | 1.02 | 1.26 | BX918559 | Transcribed locus, highly similar to NP_036250.2 CCR4 carbon catabolite repression 4-like (S. cerevisiae) (Homo sapiens) | --- | Biological rhythms |
| Ssc.11124.1.A1_at | | | 1.18 | 1.45 | BI184483 | Transcribed locus, highly similar to NP_055551.1 KIAA0101 (Homo sapiens) | --- | |

| | | | | | | | | |
|---|---|---|---|---|---|---|---|---|
| Ssc.24221.2.A1_at | -1.30 | | | -1.59 | BI181166 | Transcribed locus, highly similar to NP_035017.1 NADH dehydrogenase (ubiquinone) Fe-S protein 4 (Mus musculus) | --- | Transport |
| Ssc.27688.1.S1_at | -1.29 | | | -1.08 | BG608619 | Transcribed locus | --- | |
| Ssc.30786.1.A1_at | -1.67 | | | -1.14 | AJ657499 | Transcribed locus | --- | |
| Ssc.30752.1.S1_at | -1.66 | | | -2.15 | BX921277 | Transcribed locus | --- | |
| Ssc.31140.1.S1_at | -1.51 | | | -2.12 | BX922784 | Transcribed locus | --- | |
| Ssc.12886.1.A1_at | -1.41 | -1.09 | | | CF796076 | Transcribed locus | --- | |
| Ssc.18959.1.A1_at | -1.10 | | | -1.30 | CF365136 | Transcribed locus | --- | |
| Ssc.27195.1.A1_at | -1.36 | | | -1.21 | CN165941 | Transcribed locus | --- | |
| Ssc.29492.1.A1_at | -1.19 | | | -1.38 | CO956576 | Transcribed locus | --- | |
| Ssc.30752.2.A1_at | -1.71 | | | -2.67 | BI119523 | Transcribed locus | --- | |
| Ssc.21169.1.S1_at | -1.08 | | | -1.17 | BE032622 | Transcribed locus | --- | |
| Ssc.30637.1.A1_at | -1.05 | | | -1.67 | CO940830 | Transcribed locus | --- | |
| Ssc.25070.1.A1_at | 1.06 | | | 1.06 | CK461396 | Transcribed locus | --- | |
| Ssc.7562.1.A1_at | 1.11 | | 1.14 | | BQ599273 | Transcribed locus | --- | |
| Ssc.942.1.S1_at | 1.27 | | 1.18 | | CK465005 | Transcribed locus | --- | |
| Ssc.5182.1.A1_at | 1.28 | | | 1.19 | BF710924 | Transcribed locus | --- | |
| Ssc.25069.1.S1_at | 1.29 | | | 1.05 | CN153765 | Transcribed locus | --- | |
| Ssc.7771.1.A1_at | 1.43 | | | 1.56 | CK468060 | Transcribed locus | --- | |
| Ssc.3666.1.A1_at | 1.86 | | 1.04 | | CK459289 | Transcribed locus | --- | |
| Ssc.25948.1.S1_at | 1.92 | | 1.52 | | BX925423 | Transcribed locus | --- | |
| Ssc.12680.1.A1_at | | -1.15 | | -1.30 | BI403263 | Transcribed locus | --- | |
| Ssc.14230.1.A1_at | | -1.05 | | -1.03 | CF789095 | Transcribed locus | --- | |
| Ssc.7990.1.A1_at | | -1.05 | | -1.27 | CN166387 | Transcribed locus | --- | |
| Ssc.4081.1.A1_at | | -1.02 | | -1.10 | BQ601662 | Transcribed locus | --- | |
| Ssc.18253.1.S1_at | | | -1.16 | -1.20 | CF179697 | Transcribed locus | --- | |
| Ssc.19089.1.A1_at | | | -1.15 | -1.38 | CF367800 | Transcribed locus | --- | |
| Ssc.9039.1.A1_at | | | -1.04 | -1.24 | CF366256 | Transcribed locus | --- | |
| Ssc.5799.1.S1_at | | | -1.04 | -1.78 | BF709288 | Transcribed locus | --- | |
| Ssc.8893.1.A1_at | | | 1.18 | 1.15 | BF708608 | Transcribed locus | --- | |
| Ssc.9376.1.A1_at | | | 1.31 | 1.08 | BF710373 | Transcribed locus | --- | |
| Ssc.23778.3.S1_at | -1.49 | | | | BP167798 | prostate transmembrane protein, androgen induced 1 | CR956367.2 | Ubl conjugation pathway |
| Ssc.383.1.S1_at | -1.43 | | | | NM_213792.1 | odd homeobox 1 protein | OB1 | Transcription, transcription regulation |

| | | | | | | | | | |
|---|---|---|---|---|---|---|---|---|---|
| Ssc.23778.2.S1_at | -1.35 | | | | BX674651 | prostate transmembrane protein, androgen induced 1 | CR956367.2 | Ubl conjugation pathway |
| Ssc.3920.1.S1_at | -1.31 | | | | CN160546 | prostate transmembrane protein, androgen induced 1 | CR956367.2 | Ubl conjugation pathway |
| Ssc.23778.1.S1_at | -1.10 | | | | BE031847 | prostate transmembrane protein, androgen induced 1 | CR956367.2 | Ubl conjugation pathway |
| Ssc.14246.1.S1_at | -1.09 | | | | BX667508 | gelsolin | LOC396874 | Cytoplasm, secreted |
| Ssc.6966.2.S1_a_at | -1.03 | | | | AB031957.1 | cyclin-dependent kinase inhibitor 1B (p27, Kip1) | CDKN1B | Cell cycle, protein kinase inhibitor |
| Ssc.15841.1.S1_at | -1.01 | | | | AJ316066.1 | cyclin-dependent kinase inhibitor 2B (p15, inhibits CDK4) | CDKN2B | Cell cycle, kinase activity |
| Ssc.15893.1.S1_at | -1.00 | | | | BX676059 | sarcolipin | SLN | Regulation of calcium ion transport |
| Ssc.27187.1.S1_at | 1.00 | | | | CN160280 | similar to spindle and KT associated 1 | LOC100157355 | |
| Ssc.15588.1.S1_at | 1.01 | | | | AF085482.1 | insulin-like growth factor binding protein 3 | IGFBP3 | Regulation of apoptosis, cell growth |
| Ssc.15824.1.S1_at | 1.04 | | | | BG382957 | karyopherin alpha 2 | KPNA2 | Binding, protein transporter activity |
| Ssc.14182.1.A1_at | 1.05 | | | | CK451027 | Cyclin B2 | CCNB2 | Cell cycle, cell division |
| Ssc.16341.1.S1_at | 1.06 | | | | BP447300 | prostaglandin-endoperoxide synthase 1 | PTGS1 | Dioxygenase, oxidoreductase, peroxidase |
| Ssc.10536.1.S1_at | 1.08 | | | | CF791997 | apoptosis-related protein | PNAS-5 | Metal ion binding, protein binding |
| Ssc.11630.1.S1_at | 1.10 | | | | CK449997 | pituitary tumor-transforming 1 | PTTG1 | Cell cycle, cell division, mitosis, chromosome partition, DNA repair, DNA damage |
| Ssc.28124.1.A1_at | 1.11 | | | | CN162886 | Aurora kinase A | CR956640.2 | Cell cycle, cell division, mitosis |
| Ssc.873.1.S1_at | 1.12 | | | | AJ687786 | cell division cycle 2, G1 to S and G2 to M | CDC2 | ATP binding, protein kinase activity |
| Ssc.27540.2.S1_at | 1.14 | | | | CN166809 | similar to ubiquitin-conjugating enzyme E2C | LOC100153133 | Ubl conjugation pathway |
| Ssc.115.1.S1_s_at | 1.27 | | | | X60677.1 | heme oxygenase (decycling) 1 | HMOX1 | Oxidoreductase |
| Ssc.1466.1.A1_at | 1.27 | | | | CN160915 | aurora kinase A | CR956640.2 | Cell cycle, cell division, mitosis |
| SscAffx.8.1.S1_s_at | 1.32 | | | | NM_001005154.1 | v-myc myelocytomatosis viral oncogene homolog (avian) | MYC | Transcription, transcription regulation |
| Ssc.3139.1.A1_at | | -1.42 | | | CK456262 | Regulator of G-protein signaling 2, 24kDa | RGS2 | Signal transducer activity |
| Ssc.7314.1.A1_at | | | 1.03 | | NM_214321.1 | prostaglandin G/H synthase-2 | PGHS-2 | Oxidation reduction, response to oxidative stress |
| Ssc.17286.1.A1_at | | | 1.10 | | CK460825 | BTG family, member 2 | BTG2 | |
| Ssc.9075.1.A1_at | | | 1.44 | | NM_213880.1 | C-JUN protein | C-JUN | Transcription, transcription regulation |
| Ssc.10884.1.A1_at | | | | -2.01 | BF713431 | similar to interferon activated gene 203 | LOC100156073 | |
| Ssc.8516.1.A1_at | | | | -1.96 | NM_214389.1 | glutathione S-transferase | GSTA2 | Transferase |
| Ssc.15926.1.S1_at | | | | -1.66 | NM_214148.1 | epithelial chloride channel protein | AECC | Calcium transport, ion transport |
| Ssc.12.1.S1_at | | | | -1.45 | NM_213992.1 | sulfotransferase family 1E, estrogen-preferring, member 1 | SULT1E1 | Transferase |
| Ssc.153.1.S1_at | | | | -1.25 | NM_214044.1 | dihydropyrimidine dehydrogenase | DPYD | Oxidoreductase |
| Ssc.5604.1.S1_at | | | | -1.18 | CN154806 | protein S (alpha) | PROS1 | Calcium ion binding |
| Ssc.11422.1.A1_at | | | | -1.07 | BI182449 | similar to phospholipase A1 member A | LOC100154368 | Lipid degradation, phospholipase A1 activity |

| | | | | | | | | |
|---|---|---|---|---|---|---|---|---|
| Ssc.27786.1.S1_at | | | | 1.02 | BG384060 | Fatty acid binding protein 4, adipocyte | FABP4 | Transport, lipid binding |
| Ssc.10237.1.S1_at | | | | 1.05 | AJ301200 | similar to heat shock 27kDa protein 8 | LOC100155338 | Stress response |
| SscAffx.11.1.S1_a_at | | | | 1.07 | NM_214448.1 | involucrin | IVL | Keratinization |
| Ssc.314.1.S1_at | | | | 1.12 | NM_214107.1 | adrenomedullin | ADM | Hormone activity |
| Ssc.27786.2.S1_at | | | | 1.38 | CO994913 | CD74 antigen | CD74 | MHC class II protein binding, immune response, antigen processing and presentation |
| Ssc.26709.1.S1_at | -1.72 | | | | CN159962 | Transcribed locus, moderately similar to XP_224544.2 similar to EBV-induced G protein-coupled receptor 2 (EBI2) (Rattus norvegicus) | --- | Adaptive immunity, immunity |
| Ssc.17780.1.S1_s_at | -1.66 | | | | AB087911.1 | Transcribed locus, weakly similar to XP_135339.1 similar to RIKEN cDNA A430107P09 gene (Mus musculus) | --- | |
| Ssc.22037.1.S1_at | -1.52 | | | | CN160898 | Transcribed locus, moderately similar to NP_006753.1 Lysosomal-associated multispanning membrane protein-5 (Homo sapiens) | --- | Transport |
| Ssc.12171.1.A1_at | -1.23 | | | | BQ603723 | Transcribed locus, highly similar to XP_376328.1 family with sequence similarity 13, member A1 (Homo sapiens) | --- | |
| Ssc.22620.1.S1_at | -1.18 | | | | BX666072 | Transcribed locus, moderately similar to NP_001538.3 interferon-inducec protein with tetratricopeptide repeats 2 (Homo sapiens) | --- | Binding |
| Ssc.28517.2.S1_at | -1.16 | | | | BI346421 | Transcribed locus, highly similar to NP_071302.2 dual specificity phosphatase 10 (Mus musculus) | --- | Hydrolase |
| Ssc.20515.1.S1_at | -1.14 | | | | BG382428 | Transcribed locus, highly similar to NP_004096.2 EGF-containing fibulir like extracellular matrix protein 1 (Homo sapiens) | --- | Calcium ion binding, protein binding |
| Ssc.3648.1.S1_a_at | -1.13 | | | | F23133 | Transcribed locus, moderately similar to NP_060765.1 hypothetical protein FLJ11000 (Homo sapiens) | --- | |
| Ssc.15069.1.S1_at | -1.11 | | | | BI345060 | Transcribed locus, moderately similar to NP_570952.1 cytochrome P450, family 4, subfamily f, polypeptide 13 (Mus musculus) | --- | Monooxygenase, oxidoreductase |
| Ssc.26224.1.S1_at | -1.10 | | | | BX917028 | Transcribed locus, highly similar to NP_064567.2 transmembrane, prostate androgen induced RNA (Homo sapiens) | CR956367.2 | Ubl conjugation pathway |
| Ssc.18862.1.A1_at | -1.10 | | | | CF368734 | Transcribed locus, moderately similar to XP_221535.2 similar to downregulated in ovarian cancer 1 (Rattus norvegicus) | --- | |
| Ssc.24221.1.S1_at | -1.02 | | | | CK451013 | Transcribed locus, highly similar to NP_035017.1 NADH dehydrogenase (ubiquinone) Fe-S protein 4 (Mus musculus) | --- | Transport, NADH dehydrogenase activity |
| Ssc.1313.1.A1_at | 1.01 | | | | CK464624 | Transcribed locus, moderately similar to NP_077001.1 XTP3-transactivated protein A (Homo sapiens) | --- | Hydrolase |
| Ssc.21959.1.S1_at | 1.01 | | | | CN158067 | Transcribed locus, highly similar to NP_644802.1 cysteinyl-tRNA synthetase (Homo sapiens) | --- | Protein biosynthesis, ligase |
| Ssc.23822.1.S1_a_at | 1.02 | | | | CN161582 | Transcribed locus, highly similar to NP_001354.1 dyskeratosis congenita 1, dyskerin (Homo sapiens) | --- | Isomerase, ribonucleoprotein |

| | | | | | | | | |
|---|---|---|---|---|---|---|---|---|
| Ssc.5041.1.A1_at | 1.03 | | | | CN154405 | Transcribed locus, moderately similar to NP_006161.1 nucleolar protein 1, 120kDa (Homo sapiens) | --- | Methyltransferase, transferase |
| Ssc.5821.1.S1_at | 1.04 | | | | CK462319 | Transcribed locus, moderately similar to NP_003026.1 TAL1 (SCL) interrupting locus (Homo sapiens) | --- | Developmental protein |
| Ssc.23877.1.S1_at | 1.05 | | | | BP165838 | Transcribed locus, highly similar to NP_001228.1 cyclin A2 (Homo sapiens) | --- | Cell cycle, cell division, mitosis |
| Ssc.24422.1.S1_at | 1.06 | | | | BE235697 | Transcribed locus, highly similar to NP_055316.1 ubiquitin-conjugating enzyme E2S (Homo sapiens) | --- | Cell cycle, cell division, Ubl conjugation pathway |
| Ssc.3994.1.S1_at | 1.06 | | | | BF713387 | Transcribed locus, moderately similar to NP_115722.1 hypothetical protein MGC13096 (Homo sapiens) | --- | |
| Ssc.5401.1.S1_at | 1.06 | | | | BF190854 | Transcribed locus, highly similar to XP_375911.1 KIAA0186 gene product (Homo sapiens) | --- | |
| Ssc.22206.1.S1_at | 1.07 | | | | CK463608 | Transcribed locus, highly similar to NP_055606.1 maternal embryonic leucine zipper kinase (Homo sapiens) | --- | Kinase, transferase |
| Ssc.28512.1.S1_at | 1.09 | | | | BX672980 | Transcribed locus, moderately similar to NP_071741.2 chromosome condensation protein G (Homo sapiens) | --- | Cell cycle, cell division, mitosis, DNA condensation |
| Ssc.7195.1.A1_at | 1.09 | | | | BQ598156 | Transcribed locus, moderately similar to NP_004327.1 BUB1 budding uninhibited by benzimidazoles 1 homolog (yeast) (Homo sapiens) | --- | Apoptosis, cell cycle, cell division, mitosis, chromosome partition |
| Ssc.12475.1.A1_at | 1.11 | | | | CF364971 | Transcribed locus, highly similar to XP_222105.2 similar to expressed sequence AI480570 (Rattus norvegicus) | --- | |
| Ssc.24183.1.S1_at | 1.12 | | | | CK456980 | Transcribed locus, weakly similar to XP_341696.1 similar to RIKEN cDNA 2610510J17 (Rattus norvegicus) | --- | |
| Ssc.14117.1.A1_at | 1.12 | | | | CK450499 | Transcribed locus, moderately similar to NP_631899.1 novel 58.3 KDA protein (Homo sapiens) | --- | Intracellular signaling cascade |
| Ssc.11995.1.A1_at | 1.14 | | | | CB476520 | Transcribed locus, moderately similar to NP_006627.1 methylene tetrahydrofolate dehydrogenase (NAD+ dependent), methenyltetrahydrofolate cyclohydrolase (Homo sapiens) | --- | Hydrolase, oxydoreductase |
| Ssc.26899.1.A1_at | 1.15 | | | | CN162826 | Transcribed locus, weakly similar to NP_060674.2 cytoskeleton associated protein 2 (Homo sapiens) | --- | Apoptosis, cell cycle |
| Ssc.21217.1.A1_at | 1.16 | | | | CF792040 | Transcribed locus, moderately similar to XP_372192.2 Similar to RIKEN cDNA 2700049P18 gene (Homo sapiens) | --- | |
| Ssc.7594.1.A1_at | 1.25 | | | | CN165502 | Transcribed locus, highly similar to NP_060839.1 DEP domain containing 1B (Homo sapiens) | --- | Intracellular signaling cascade |
| Ssc.11108.1.A1_at | 1.29 | | | | CF792943 | Transcribed locus, moderately similar to NP_000295.1 peripheral myelin protein 22 (Homo sapiens) | --- | |
| Ssc.1986.1.S1_at | 1.35 | | | | CK452098 | Transcribed locus, moderately similar to NP_542158.1 prostaglandin-endoperoxide synthase 1 (prostaglandin GH synthase and cyclooxygenase) (Homo sapiens) | --- | Dioxygenase, oxidoreductase, peroxidase |
| Ssc.22545.1.A1_at | 1.37 | | | | CK449996 | Transcribed locus, highly similar to NP_060255.2 hypothetical protein FLJ20364 (Homo sapiens) | --- | Cell cycle, cell division, mitosis |
| Ssc.26568.1.A1_s_at | 1.42 | | | | CN153199 | Transcribed locus, weakly similar to NP_149020.1 cyclin B3 (Homo sapiens) | --- | Cell cycle, cell division, meiosis |

| | | | | | | | | |
|---|---|---|---|---|---|---|---|---|
| Ssc.19382.1.A1_at | 1.48 | | | | CF180618 | Transcribed locus, highly similar to NP_004554.1 phosphoenolpyruvate carboxykinase 2 (mitochondrial) (Homo sapiens) | --- | Decarboxylase, lyase |
| Ssc.11609.1.A1_at | 1.52 | | | | BI182003 | Transcribed locus, highly similar to NP_001664.2 asparagine synthetase (Homo sapiens) | --- | Ligase |
| Ssc.18999.1.A1_at | | | -1.09 | | CO940748 | Transcribed locus, moderately similar to NP_848599.2 hypothetical protein FLJ40427 (Homo sapiens) | --- | Ubl conjugation pathway |
| Ssc.16460.1.S1_at | | | 1.13 | | CB285513 | Transcribed locus, highly similar to NP_001955.1 early growth response 1 (Homo sapiens) | --- | Transcription, transcription regulation |
| Ssc.24986.1.S1_at | | | | -1.64 | CK463807 | Transcribed locus, highly similar to NP_000680.2 aldehyde dehydrogenase 1 family, member A1 (Homo sapiens) | --- | Oxidoreductase |
| Ssc.17353.1.S1_at | | | | -1.58 | CK459510 | Transcribed locus, moderately similar to NP_031712.1 complement component factor i (Mus musculus) | --- | |
| Ssc.5453.1.A1_at | | | | -1.49 | CK465332 | Transcribed locus, moderately similar to NP_036545.1 glutaminyl-peptide cyclotransferase (glutaminyl cyclase) (Homo sapiens) | --- | Acyltransferase, transferase |
| Ssc.5557.1.S1_at | | | | -1.45 | CN153429 | Transcribed locus, moderately similar to NP_780750.1 RIKEN cDNA 9430086K21 gene (Mus musculus) | --- | |
| Ssc.19980.1.S1_at | | | | -1.35 | BX675506 | Transcribed locus, weakly similar to NP_055080.1 a disintegrin and metalloproteinase domain 28 (Homo sapiens) | --- | Hydrolase, protease, metalloprotease |
| Ssc.24974.1.S1_at | | | | -1.32 | BE234949 | Transcribed locus, highly similar to XP_223684.2 similar to Peroxisomal membrane protein PEX13 (Peroxin-13) (Rattus norvegicus) | --- | Protein transport, transport |
| Ssc.6595.1.A1_at | | | | -1.30 | BQ599735 | Transcribed locus, moderately similar to NP_080498.1 DNA segment, Chr 3, ERATO Doi 789, expressed (Mus musculus) | --- | |
| Ssc.11246.1.A1_at | | | | -1.23 | BI181165 | Transcribed locus, moderately similar to XP_346818.1 Rattus norvegicus LOC360432 gene | --- | |
| Ssc.25962.1.S1_at | | | | -1.18 | BX925692 | Transcribed locus, moderately similar to NP_008825.3 flavin containing monooxygenase 3 (Homo sapiens) | --- | Monooxygenase, oxidoreductase |
| Ssc.14318.1.S1_at | | | | -1.14 | CK449951 | Transcribed locus, highly similar to NP_789794.1 Bardet-Biedl syndrome 7 (Homo sapiens) | --- | Protein binding |
| Ssc.12504.1.A1_at | | | | -1.09 | CK465051 | Transcribed locus, weakly similar to NP_919043.1 DNA segment, Chr 12, ERATO Doi 647, expressed (Mus musculus) | --- | |
| Ssc.10110.1.A1_at | | | | -1.08 | BQ599693 | Transcribed locus, highly similar to NP_758527.1 Meis1, myeloid ecotropic viral integration site 1 homolog 2 (mouse) (Homo sapiens) | --- | Regulation of transcription, DNA dependent |
| Ssc.11477.3.A1_at | | | | -1.07 | CF794867 | Transcribed locus, highly similar to NP_115938.1 IMP2 inner mitochondrial membrane protease-like (S. cerevisiae) (Homo sapiens) | --- | Hydrolase, protease |
| Ssc.7785.1.S1_at | | | | -1.06 | CK456652 | Transcribed locus, moderately similar to NP_443164.1 pro-oncosis receptor inducing membrane injury gene (Homo sapiens) | --- | Receptor |
| Ssc.6425.3.A1_at | | | | -1.06 | BI399085 | Transcribed locus, moderately similar to NP_005679.1 ATP-binding cassette, sub-family C (CFTRMRP), member 5 (Homo sapiens) | --- | Transport |

| | | | | | | | | |
|---|---|---|---|---|---|---|---|---|
| Ssc.26864.1.A1_at | | | | -1.02 | CN162547 | Transcribed locus, weakly similar to XP_150149.1 RIKEN cDNA 9230113P08 gene (Mus musculus) | --- | |
| Ssc.10238.1.A1_at | | | | -1.02 | BI400310 | Transcribed locus, moderately similar to NP_035519.1 solute carrier family 12, member 1 (Mus musculus) | --- | |
| Ssc.13226.1.A1_at | | | | -1.02 | BQ604865 | Transcribed locus, moderately similar to NP_113646.1 B aggressive lymphoma gene (Homo sapiens) | --- | Transferase |
| Ssc.27912.1.S1_at | | | | -1.01 | CF365868 | Transcribed locus, highly similar to XP_114432.2 zinc finger protein 608 (Homo sapiens) | --- | Zinc ion binding |
| Ssc.11164.2.S1_a_at | | | | 1.02 | CO953604 | Transcribed locus, highly similar to NP_445980.1 DNA polymerase gamma (Rattus norvegicus) | --- | DNA-directed DNA polymerase, nucleotidyltransferase, transferase |
| Ssc.8947.1.S1_at | | | | 1.03 | BX667843 | Transcribed locus, moderately similar to NP_003661.1 basic helix-loop-helix domain containing, class B, 2 (Homo sapiens) | --- | |
| Ssc.17230.1.A1_at | | | | 1.04 | CK460624 | Transcribed locus, weakly similar to XP_343961.1 similar to Da1-6 (Rattus norvegicus) | --- | Actin binding |
| Ssc.21184.1.S1_at | | | | 1.06 | CN155420 | Transcribed locus, highly similar to NP_032272.1 hippocampus abundant gene transcript 1 (Mus musculus) | --- | Transport |
| Ssc.25920.1.S1_at | | | | 1.08 | BX925015 | Transcribed locus, highly similar to XP_213765.2 similar to CCT (chaperonin containing TCP-1) zeta subunit (Rattus norvegicus) | --- | ATP binding, protein folding |
| Ssc.11519.2.A1_at | | | | 1.10 | BI183297 | Transcribed locus, moderately similar to NP_075813.1 pellino 1 (Mus musculus) | --- | Protein binding, ubiquitin-protei ligase activity |
| Ssc.11519.1.A1_at | | | | 1.16 | BI185881 | Transcribed locus, moderately similar to NP_075813.1 pellino 1 (Mus musculus) | --- | Protein binding, ubiquitin-protei ligase activity |
| Ssc.5396.1.S1_at | | | | 1.23 | CF792567 | Transcribed locus, highly similar to NP_003197.2 transcription factor 21 (Homo sapiens) | --- | Transcription, transcription regulation |
| Ssc.7524.1.A1_at | -1.30 | | | | BQ599075 | Transcribed locus | --- | |
| Ssc.27680.1.S1_at | -1.28 | | | | AW480248 | Transcribed locus | --- | |
| Ssc.7637.2.S1_at | -1.26 | | | | BI339207 | Transcribed locus | --- | |
| Ssc.29750.1.A1_at | -1.25 | | | | CO993759 | Transcribed locus | --- | |
| Ssc.4664.1.S1_at | -1.25 | | | | BM482000 | Transcribed locus | --- | |
| Ssc.2274.1.A1_at | -1.23 | | | | BE232665 | Transcribed locus | --- | |
| Ssc.27042.1.A1_at | -1.22 | | | | CN165949 | Transcribed locus | --- | |
| Ssc.30871.1.A1_at | -1.17 | | | | BI118962 | Transcribed locus | --- | |
| Ssc.18929.1.S1_s_at | -1.14 | | | | CF366027 | Transcribed locus | --- | |
| Ssc.6382.1.A1_at | -1.13 | | | | BI343750 | Transcribed locus | --- | |
| Ssc.26680.1.A1_at | -1.10 | | | | CN154736 | Transcribed locus | --- | |
| Ssc.4272.1.A1_at | -1.10 | | | | BQ599843 | Transcribed locus | --- | |
| Ssc.17387.1.A1_at | -1.06 | | | | CO948677 | Transcribed locus | --- | |
| Ssc.10466.1.A1_at | -1.09 | | | | BQ602626 | Transcribed locus | --- | |

| | | | | | | | | |
|---|---|---|---|---|---|---|---|---|
| Ssc.3271.1.A1_at | -1.03 | | | | BF703659 | Transcribed locus | --- | |
| Ssc.19413.1.A1_at | -1.02 | | | | CF360057 | Transcribed locus | --- | |
| Ssc.6697.1.S1_at | -1.01 | | | | CO954523 | Transcribed locus | --- | |
| Ssc.5648.1.A1_at | -1.01 | | | | BF702975 | Transcribed locus | --- | |
| Ssc.30194.1.A1_at | -1.00 | | | | CN162805 | Transcribed locus | --- | |
| Ssc.21700.1.A1_at | -1.00 | | | | CF792064 | Transcribed locus | --- | |
| Ssc.24758.1.A1_at | 1.03 | | | | CK466750 | Transcribed locus | --- | |
| Ssc.18028.1.A1_at | 1.03 | | | | CF180509 | Transcribed locus | --- | |
| Ssc.9504.1.A1_at | 1.04 | | | | BF710717 | Transcribed locus | --- | |
| Ssc.18907.1.A1_at | 1.05 | | | | CF365630 | Transcribed locus | --- | |
| Ssc.13591.1.A1_at | 1.07 | | | | BQ602801 | Transcribed locus | --- | |
| Ssc.3088.1.S1_at | 1.07 | | | | CN166193 | Transcribed locus | --- | |
| Ssc.30351.1.A1_at | 1.12 | | | | CO990628 | Transcribed locus | --- | |
| Ssc.21130.1.S1_at | 1.13 | | | | CK449422 | Transcribed locus | --- | |
| Ssc.7546.1.A1_at | 1.14 | | | | BQ599193 | Transcribed locus | --- | |
| Ssc.5863.1.S1_at | 1.14 | | | | BI184061 | Transcribed locus | --- | |
| Ssc.6097.1.S1_a_at | 1.15 | | | | BX672990 | Transcribed locus | --- | |
| Ssc.30436.1.A1_at | 1.16 | | | | CO991902 | Transcribed locus | --- | |
| Ssc.27421.1.A1_at | 1.16 | | | | CN160251 | Transcribed locus | --- | |
| Ssc.27298.1.A1_at | 1.17 | | | | CK464598 | Transcribed locus | --- | |
| Ssc.24584.1.S1_at | 1.18 | | | | CK460583 | Transcribed locus | --- | |
| Ssc.24459.1.S1_at | 1.27 | | | | CK457302 | Transcribed locus | --- | |
| Ssc.13547.1.A1_at | 1.33 | | | | BQ602672 | Transcribed locus | --- | |
| Ssc.29855.1.A1_at | 1.37 | | | | CO937870 | Transcribed locus | --- | |
| Ssc.31016.1.A1_at | 1.43 | | | | CO949604 | Transcribed locus | --- | |
| Ssc.25317.1.S1_at | 1.53 | | | | BX914997 | Transcribed locus | --- | |
| Ssc.29281.1.A1_at | 1.68 | | | | CO953055 | Transcribed locus | --- | |
| Ssc.9440.1.A1_at | 2.30 | | | | CN069729 | Transcribed locus | --- | |
| Ssc.3509.1.S1_at | | -1.35 | | | BE235252 | Transcribed locus | --- | |
| Ssc.6294.2.S1_at | | -1.25 | | | BI340023 | Transcribed locus | --- | |
| Ssc.7834.1.A1_at | | -1.02 | | | BQ599559 | Transcribed locus | --- | |
| Ssc.21884.1.A1_at | | 1.10 | | | CF794971 | Transcribed locus | --- | |
| Ssc.27410.1.S1_at | | | -1.22 | | CN159453 | Transcribed locus | --- | |

| | | | | | | | | | |
|---|---|---|---|---|---|---|---|---|---|
| Ssc.22608.1.S1_at | | | -1.14 | | BX667042 | Transcribed locus | --- | |
| Ssc.18307.1.A1_at | | | -1.08 | | BQ597547 | Transcribed locus | --- | |
| Ssc.22521.1.A1_at | | | -1.05 | | CK465789 | Transcribed locus | --- | |
| Ssc.19629.2.S1_s_at | | | 1.01 | | BQ603077 | Transcribed locus | --- | |
| Ssc.3411.1.A1_at | | | 1.05 | | CK461446 | Transcribed locus | --- | |
| Ssc.16466.1.A1_at | | | 1.15 | | CK459813 | Transcribed locus | --- | |
| Ssc.13423.1.A1_at | | | 1.19 | | BI404586 | Transcribed locus | --- | |
| Ssc.8436.1.S1_at | | | 1.19 | | BF710666 | Transcribed locus | --- | |
| Ssc.4643.1.A1_at | | | 1.35 | | BF712417 | Transcribed locus | --- | |
| Ssc.22550.1.A1_at | | | 1.37 | | CO943105 | Transcribed locus | --- | |
| Ssc.9607.1.A1_at | | | 1.53 | | BF711037 | Transcribed locus | --- | |
| Ssc.3189.1.A1_at | | | 1.60 | | BQ600803 | Transcribed locus | --- | |
| Ssc.30916.1.A1_at | | | | -1.50 | CO947301 | Transcribed locus | --- | |
| Ssc.14075.1.A1_at | | | | -1.46 | CK459115 | Transcribed locus | --- | |
| Ssc.6928.1.A1_at | | | | -1.45 | CO989436 | Transcribed locus | --- | |
| Ssc.31189.1.S1_at | | | | -1.31 | CO952477 | Transcribed locus | --- | |
| Ssc.23764.1.S1_at | | | | -1.30 | BG895329 | Transcribed locus | --- | |
| Ssc.19337.1.A1_at | | | | -1.29 | BM190087 | Transcribed locus | --- | |
| Ssc.30063.1.A1_at | | | | -1.24 | CO951943 | Transcribed locus | --- | |
| Ssc.7558.1.A1_at | | | | -1.24 | BQ599267 | Transcribed locus | --- | |
| Ssc.26005.1.S1_at | | | | -1.24 | BX926252 | Transcribed locus | --- | |
| Ssc.22362.1.A1_at | | | | -1.23 | CF792366 | Transcribed locus | --- | |
| Ssc.29231.1.A1_at | | | | -1.21 | CO952377 | Transcribed locus | --- | |
| Ssc.17861.1.A1_at | | | | -1.20 | BI118294 | Transcribed locus | --- | |
| Ssc.21026.1.S1_at | | | | -1.19 | CD572509 | Transcribed locus | --- | |
| Ssc.10673.1.A1_at | | | | -1.16 | BI404740 | Transcribed locus | --- | |
| Ssc.22377.1.A1_at | | | | -1.15 | CF792421 | Transcribed locus | --- | |
| Ssc.9039.2.S1_at | | | | -1.15 | CF364838 | Transcribed locus | --- | |
| Ssc.14415.1.A1_at | | | | -1.15 | BQ602223 | Transcribed locus | --- | |
| Ssc.5187.1.S1_at | | | | -1.14 | BI405042 | Transcribed locus | --- | |
| Ssc.8238.1.S1_at | | | | -1.13 | BF702474 | Transcribed locus | --- | |
| Ssc.25145.1.S1_at | | | | -1.12 | CK465844 | Transcribed locus | --- | |
| Ssc.7893.1.A1_at | | | | -1.12 | BI404005 | Transcribed locus | --- | |

| | | | | | | | | |
|---|---|---|---|---|---|---|---|---|
| Ssc.23126.1.S1_at | | | | -1.11 | BX674432 | Transcribed locus | --- | |
| Ssc.7755.1.A1_at | | | | -1.11 | CF792275 | Transcribed locus | --- | |
| Ssc.24178.1.A1_at | | | | -1.11 | CK457006 | Transcribed locus | --- | |
| Ssc.23368.1.A1_at | | | | -1.10 | BQ599629 | Transcribed locus | --- | |
| Ssc.26983.1.A1_at | | | | -1.09 | CN164747 | Transcribed locus | --- | |
| Ssc.29749.1.A1_at | | | | -1.09 | CO942872 | Transcribed locus | --- | |
| Ssc.13053.1.A1_at | | | | -1.08 | BQ603979 | Transcribed locus | --- | |
| Ssc.7907.1.A1_at | | | | -1.08 | BI181465 | Transcribed locus | --- | |
| Ssc.14352.1.S1_at | | | | -1.06 | AJ656944 | Transcribed locus | --- | |
| Ssc.28084.1.A1_at | | | | -1.05 | CN031521 | Transcribed locus | --- | |
| Ssc.26692.1.A1_at | | | | -1.05 | CN157023 | Transcribed locus | --- | |
| Ssc.17005.1.A1_at | | | | -1.05 | CB475105 | Transcribed locus | --- | |
| Ssc.10642.1.S1_at | | | | -1.04 | CN162548 | Transcribed locus | --- | |
| Ssc.13414.1.A1_at | | | | -1.03 | BI405191 | Transcribed locus | --- | |
| Ssc.25324.1.S1_at | | | | -1.02 | CO946796 | Transcribed locus | --- | |
| Ssc.4427.1.A1_at | | | | -1.02 | BQ598146 | Transcribed locus | --- | |
| Ssc.27465.1.S1_at | | | | 1.05 | CN160638 | Transcribed locus | --- | |
| Ssc.30870.1.A1_at | | | | 1.37 | CF790577 | Transcribed locus | --- | |
